# Translating Measures of Biological Aging to Test Effectiveness of Geroprotective Interventions: What Can We Learn from Research on Telomeres?

**DOI:** 10.3389/fgene.2017.00164

**Published:** 2017-11-22

**Authors:** Waylon J. Hastings, Idan Shalev, Daniel W. Belsky

**Affiliations:** ^1^Department of Biobehavioral Health, Pennsylvania State University, State College, PA, United States; ^2^Department of Population Health Sciences, Duke University School of Medicine, Durham, NC, United States; ^3^Center for the Study of Aging and Human Development, Duke University, Durham, NC, United States

**Keywords:** biological aging, telomere, biomarkers, aging, geroscience

## Abstract

Intervention studies in animals suggest molecular changes underlying age-related disease and disability can be slowed or reversed. To speed translation of these so-called “geroprotective” therapies to prevent age-related disease and disability in humans, biomarkers are needed that can track changes in the rate of human aging over the course of intervention trials. Algorithm methods that measure biological processes of aging from combinations of DNA methylation marks or clinical biomarkers show promise. To identify next steps for establishing utility of these algorithm-based measures of biological aging for geroprotector trials, we considered the history a candidate biomarker of aging that has received substantial research attention, telomere length. Although telomere length possesses compelling biology to recommend it as a biomarker of aging, mixed research findings have impeded clinical and epidemiologic translation. Strengths of telomeres that should be established for algorithm biomarkers of aging are correlation with chronological age across the lifespan, prediction of disease, disability, and early death, and responsiveness to risk and protective exposures. Key challenges in telomere research that algorithm biomarkers of aging must address are measurement precision and reliability, establishing links between longitudinal rates of change across repeated measurements and aging outcomes, and clarity over whether the biomarker is a causal mechanism of aging. These strengths and challenges suggest a research agenda to advance translation of algorithm-based aging biomarkers: establish validity in young-adult and midlife individuals; test responsiveness to exposures that shorten or extend healthy lifespan; and conduct repeated-measures longitudinal studies to test differential rates of change.

Biomarkers of aging are needed to advance translation of new therapies to slow aging and extend healthy lifespan or “healthspan.” Accumulating evidence suggests molecular changes that occur with aging are among the root causes of age-related disease and disability (Lopez-Otin et al., 2013; Kennedy et al., 2014). Experiments with animals show that these molecular changes can be slowed or reversed, producing increases in healthy lifespan (Fontana et al., 2014; Kaeberlein et al., 2015). Translation of these therapies, called “geroprotectors” (Moskalev et al., 2016), to extend human healthspan is increasingly plausible (Newgard and Sharpless, 2013; Longo et al., 2015; Newman et al., 2016). A barrier to translation is the challenge of measuring changes in the rate of human aging.

Unlike worms, flies, and mice, humans live too long to observe complete lifespans within individual studies. Age-related disease and disability typically develop over a period of decades spanning the second half of the human life course. Preventive interventions are therefore needed relatively early, before age-related disease becomes established (Moffitt et al., 2017). True tests of the effectiveness of such interventions will require decades of follow-up. To establish proof of concept for such long-term studies, measurements are needed that allow tests of a candidate therapy’s potential to slow the rate of human aging over short periods of time (Justice et al., 2016; Belsky et al., 2017b).

Measurement of aging biomarkers before, during, and after administration of therapy in treatment and control groups would allow for a simple test of whether the therapy showed promise to slow aging and extend healthspan. Such aging biomarkers may be within reach from new algorithms that combine information from multiple clinical parameters and dozens or hundreds of gene expression or DNA methylation measurements (Cohen et al., 2013; Hannum et al., 2013; Horvath, 2013; Levine, 2013; Weidner et al., 2014; Belsky et al., 2015; Peters et al., 2015; Sood et al., 2015). Initial epidemiologic studies of these algorithm-based biomarkers of aging indicate promise (Jylhava et al., 2017). For example, so-called “epigenetic clocks” composed of dozens or hundreds of methylation marks have been shown to predict mortality in multiple studies and have also been linked with disability, disease processes, and age-related cognitive decline (Marioni et al., 2015; Breitling et al., 2016; Chen et al., 2016; Levine et al., 2016). Research is needed to test if these new aging biomarkers can inform evaluations of candidate therapies to slow aging and extend healthspan. We consider the case of a candidate biomarker of aging for which there has been substantial research, telomere length, with the aim of deriving lessons to guide design of new studies to evaluate algorithm-based aging biomarkers.

## Telomeres as Aging Biomarkers

**Telomeric DNA functions as a cellular biological clock and a proximate cause of cellular senescence.** Telomeres are repetitive nucleoprotein regions (TTAGGG in humans) at chromosome ends which prevent end to end fusions and protect against DNA degradation. Each time a cell divides, 12–30 base-pairs of telomeric DNA are lost due to inefficiency in DNA replication machinery, a phenomena known as the “end-replication problem” (Olovnikov, 1971; Watson, 1972). Successive cell divisions, combined with processes of “wear and tear,” gradually erode telomere length (Shalev and Hastings, in press). When telomeres become critically short, cells enter a state of replicative arrest called senescence, a “vegetative state” in which the cell no longer divides, but remains metabolically active and adopts an immunogenic phenotype (di Fagagna et al., 2003). Senescent cells release signaling molecules that, while adaptive in small doses, when unchecked can lead to dysregulation or damage of surrounding tissue (Campisi, 2013). Accumulation of senescent cells is thought to be a core driver of aging-related decline in system integrity (Baker et al., 2011; Lopez-Otin et al., 2013; Akbar et al., 2016). In stem and germ cells, telomere erosion is counteracted by the enzyme telomerase, which adds telomeric repeats during cell division, decelerating erosion, and resulting senescence (Greider and Blackburn, 1985; Kim et al., 1994). In contrast, somatic cells have only low levels of telomerase and experience telomere shortening with successive divisions, eventually leading to senescence. Thus, for somatic cells, the length of the telomeric repeat region is both a marker of the biological age of the cell, reflecting time until senescence, and a mechanism of biological aging, functioning as a proximate cause of cellular senescence. For these reasons, telomere length has been proposed as a biomarker of aging (von Zglinicki and Martin-Ruiz, 2005).

**Shorter telomere length is associated with advanced chronological age, risk of age-related disease, and early mortality.** In humans, telomeres tend to be shorter in older as compared to younger individuals (Frenck et al., 1998) and, in longitudinal studies that measure telomeres in the same humans at two time points, telomeres tend to be shorter at the later measurement (Chen et al., 2011). Critically, among individuals of the same age, those with shorter telomeres more often develop chronic disease and are at increased risk for death (Ma et al., 2011; Haycock et al., 2014; D’Mello et al., 2015; Rode et al., 2015), whereas exceptionally long-lived persons and populations tend to have longer telomeres (Atzmon et al., 2010; Rehkopf et al., 2013). Important to potential use of telomeres to measure effectiveness of therapies to extend healthspan, telomere length in blood declines from early in life (Sidorov et al., 2009) and age-dependent telomere shortening can be observed well in advance of chronic disease onset (Zeichner et al., 1999; Aviv et al., 2009). Thus, telomere length has potential to provide information about aging processes before they accumulate to establish disease and disability.

**Exposures known to increase disease risk and shorten lifespan are associated with shorter telomere length.** Environmental exposures can modify telomere length. Biometric analyses using the twin design indicate that environmental factors account for substantial variation in telomere length (Bakaysa et al., 2007), and the relative proportion of telomere length variance attributable to environmental causes increases with advancing chronological age (Hjelmborg et al., 2015). Risk exposures beginning in early life, including intrauterine stress (Entringer et al., 2012; Marchetto et al., 2016), childhood maltreatment, and deprivation (Ridout et al., 2017), as well as exposures experienced later in life such as chronic psychosocial stress (Epel et al., 2004; Cherkas et al., 2006), substance abuse (Pavanello et al., 2011), and sleep deprivation (Liang et al., 2011; Carroll et al., 2016) are associated with shorter telomere length, whereas healthy lifestyle behaviors are associated with longer telomeres and increased telomerase activity (Puterman et al., 2010; Jacobs et al., 2011). Although there are few repeated-measures longitudinal analyses of risk exposures and telomere erosion (Mather et al., 2011), there is some evidence that childhood adversity and adult mental health problems are associated with faster decline in telomere length over time (Shalev, 2012; Shalev et al., 2013b, 2014). Therefore, there is at least suggestive evidence that telomeres could function as a mediator connecting risk exposures with diminished healthspan, and thus constitute a potential therapeutic target. In fact, private companies have been developed on precisely this premise to market therapies aimed at elongating telomeres or slowing their erosion (Harley et al., 2011; Hamzelou, 2017).

## Methodological and Inferential Challenges

Alongside evidence supporting telomeres as an aging biomarker, challenges related to measurement and causal inference have impeded translation. Two measurement challenges are variability in measurement approaches across studies and limited precision of many measurement methods for quantifying telomere-related cellular senescence.

Variability in measurement approaches is a challenge because it makes it hard to compare results from different analyses. There is no single technology to assay telomeres; instead, there are multiple methods that vary in their precision (Aubert et al., 2012). Even when the same method is used, variation in sample preparation can limit comparability across assays (Cunningham et al., 2013). Further, the technologies most commonly used to assay telomeres in human epidemiologic studies do not produce estimates of a fixed quantity that can be directly compared to measurements made in other labs (Aviv et al., 2011). Poor comparability of assays and low precision is one explanation advanced for the confusing phenomenon of telomere lengthening observed in longitudinal studies (Steenstrup et al., 2013), although apparent lengthening may also reflect biological processes (Bateson and Nettle, 2017; Rivera et al., 2017).

**FIGURE 1:**
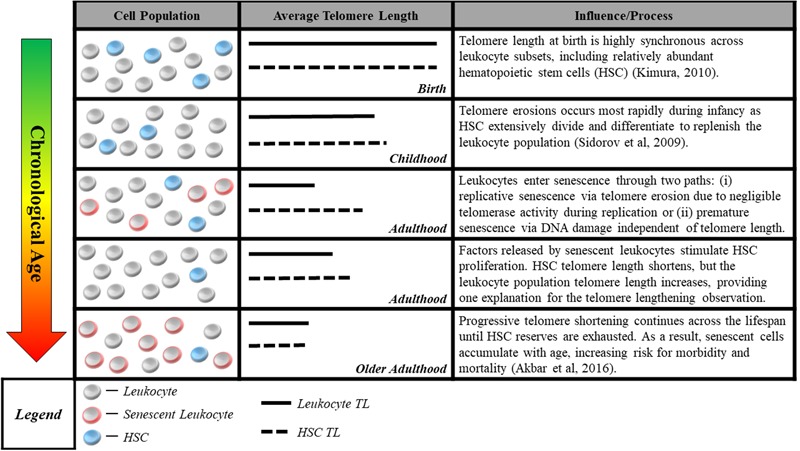
Blood sample cell composition and telomere length measurement. The figure shows aging-related changes in blood–leukocyte populations and illustrates how aging-related changes to leukocyte composition can affect telomere length measurements. Many studies report average telomere length from leukocytes, a heterogeneous class comprised of multiple distinct cell-types including granulocytes, lymphocytes, monocytes, as well as hematopoietic stem cells (HSC). Although telomere length is highly synchronous across leukocyte subsets at birth (Kimura et al., 2010), it can be significantly variable across the life course, with HSC telomeres tending to be longer than those of granulocytes and monocytes (Lasho et al., 2013). Telomere length in HSC most accurately captures the aging process, representing the organism’s capacity for self-renewal. However, only naïve, newly differentiated leukocytes have telomeres closely resembling that of HSC progenitors, while older leukocytes have shorter telomere length (Kimura et al., 2010), indicating closer proximity to senescence. This distribution of lengths resulting from leukocyte turnover makes it difficult to parse out whether differences in telomere length are attributable to blood-sample leukocyte composition or an exposure of interest. Cell-type heterogeneity can be partially corrected for, e.g., using complete blood count data (Cole, 2010), but the potential to confound telomere measurements remains a concern.

The second measurement challenge is that methods commonly used to measure telomere length in human studies do not reliably quantify cells’ proximity to senescence, the mechanism linking telomeres to aging. Quantitative polymerase chain reaction (Cawthon, 2002) and Southern blot methods (Allshire et al., 1989) assess average telomere length for a population of cells in a tissue sample. However, the telomere parameter most critical for cell viability may be the length of the single shortest telomere (Hemann et al., 2001). Thus, even the average telomere length within a single cell may be a poor proxy for that cell’s risk of short-telomere-induced senescence. This challenge is compounded by the aggregation of telomeres across cells of different types, some of which may age at different rates (Friedrich et al., 2000; Takubo et al., 2002). In addition, factors related to the tissue from which DNA is sampled may further separate telomere length measurements from the proximity to senescence of the cells measured. An example from the case of blood, the tissue most commonly assayed in human studies, is illustrated in **Figure 1**.

A central inferential challenge is determining whether shorter average telomere length in a blood sample is a cause or merely a correlate of shortened healthspan. Telomeres may be shortened by many factors, repeated cell divisions being only one (Shalev and Hastings, in press). Notably, DNA damage via oxidative cleavage (von Zglinicki, 2002) and elevated inflammatory activity (O’Donovan et al., 2011) both induce telomere erosion. These same two processes of oxidative stress and inflammation also increase with age (Khansari et al., 2009) and can cause cellular senescence (Correia-Melo et al., 2014), the main pathway by which telomeres are hypothesized to cause aging. For example, oxidative DNA damage can trigger premature cellular senescence independent of telomere length (Serrano et al., 1997; Chen et al., 2001). In turn, senescent cells release inflammatory factors as part of the so-called senescence-associated secretory phenotype (Rodier et al., 2009). Thus, telomere biology leaves open the possibility that shorter telomere length can be both a cause and a correlate of aging-related cellular senescence. Among the strongest evidence for telomeres as a casual influence on aging are the so-called “telomere syndromes” in which deficient telomere biology produces a progeroid (fast-aging) phenotype (Armanios and Blackburn, 2012). Here too, though, uncertainty remains, e.g., mice, which have longer telomeres than humans, can still develop progeroid syndromes without concurrent telomere shortening (Garinis et al., 2008).

Telomere epidemiology is similarly equivocal. The strongest nonexperimental design for testing causality of telomere length–healthspan associations in humans is Mendelian randomization (Evans and Davey Smith, 2015), which uses genetic variants linked with telomere length to disentangle telomere effects on healthspan from reverse causation. So far findings from this design are mixed and include evidence that longer telomeres may increase risk for some cancers (Rode et al., 2015; Zhan et al., 2015; Zhang et al., 2015; Haycock et al., 2017). Longitudinal repeated-measures studies of telomere length change with advancing age are still few in number and most are limited to only two time points. In these studies, average telomere length does shorten with advancing chronological age (Vaupel, 2010; Muezzinler et al., 2013). However, the rate of change is positively correlated with baseline telomere length (Nordfjall et al., 2009), consistent with regression to the mean (Verhulst et al., 2013). One explanation is increased measurement error in two-time-point analyses of change (Bereiter, 1963). Whether the rate of telomere shortening can forecast disease, disability, or earlier death remains uncertain (Martin-Ruiz et al., 2005; Boonekamp et al., 2013; Arai et al., 2015). Three-time-point studies may clarify findings. Another reason may be mortality selection (Yashin et al., 1985). In studies of older adults, participation in follow-up assessments may vary as a function of biological aging. Fast-aging people may be more likely to die or be lost to follow-up for other reasons. As a result, those persons observed at a second telomere measurement interval may reflect increased “healthy participant” bias.

## A Research Agenda to Advance Translation of Algorithm-Based Measures of Biological Aging

In sum telomere length has strengths as an aging biomarker, but there are important challenges to implementation that leave translational potential uncertain. These strengths and challenges suggest a research agenda to advance development of new algorithm-based aging biomarkers. Two constants in aging biomarker research are that putative biomarkers should correlate with chronological age and should account for age-dependent increases in risk for morbidity and mortality (Butler et al., 2004; Sprott, 2010). In addition to these criteria, telomere research suggests at least three additional objectives:

(1) Establish validity across the lifespan. A primary strength of telomeres as a biomarker of aging is that age-related telomere shortening occurs across the life course. This matches theoretical models of aging as a lifelong process (Kirkwood, 2005). Epigenetic clocks also have this property (Horvath, 2013). Some algorithms combining information from clinical biomarkers already detect variation by young adulthood (Belsky et al., 2015), but their potential utility in children remains untested. Evidence for biological embedding of early-life stress resulting in physiological dysregulation during childhood (Danese and McEwen, 2012) suggests that such studies have promise. But whether the same clinical biomarker algorithms that measure aging in adults can provide similar information prior to puberty is unknown. Of greatest importance is the ability of an algorithm to track aging processes from young adulthood, when interventions to slow aging may first be administered.
(2) Test responsiveness to exposures known to shorten or extend healthy lifespan. A second strength of telomeres as an aging biomarker is that exposures that predict shorter healthspan are associated with shorter telomeres well before onset of age-related disease, although this finding needs further confirmation from longitudinal studies (Shalev et al., 2013a). There is preliminary evidence that epigenetic clocks are accelerated by perinatal risk exposures (Simpkin et al., 2016) and that a range of early life risks forecast accelerated aging measured by clinical biomarker algorithms (Belsky et al., 2017a). Evidence on how health behaviors and other risk and protective factors may affect biological aging measures is also beginning to accumulate (Horvath et al., 2016; Brown et al., 2017; Dugue et al., 2017). An important next step is to test whether measures of biological aging mediate associations between risk and protective factors and disease, disability, and death.
(3) Conduct longitudinal repeated measures studies to test differential rates of change. Longitudinal repeated measures studies are needed to determine if cross-sectional measurements suggesting accelerated biological aging in fact reflect differential rates of change or instead differences established early in development. Just as critical, as Ingram et al. (2001) noted more than a decade ago, to be a valid measure of aging, differential rates of change must forecast the adverse outcomes of aging that geroprotective intervention will aim to delay or prevent. A premium should be placed on conducting longitudinal studies of three or more repeated measurements. Measurement error can be inflated in two-time-point studies. To distinguish true change from measurement error, at least three time points are needed.

Standardization of measurement technology for algorithm-based biomarkers of aging can help to achieve these objectives. In telomere research, lack of comparability of measurements across labs has slowed progress. Differences in assay technology and analysis methods are both issues. As an example of the benefits of standardization, epigenetic clock research achieved rapid progress partly because many different labs used the same assay technology and investigators publicly disseminated software to implement analysis. The Horvath lab’s website interface to aid researchers in computing epigenetic clocks in their own data^1^ is a model to be emulated. Because of variation in assay technology, it is unclear if the same kind of standardization is possible for transcriptomic, proteomic, or metabolomic clocks. But for clinical biomarker algorithms, an automated system is possible, e.g., the aging.ai tool^2^ (Putin et al., 2016). Scaling such tools to allow researchers to upload and score entire datasets or to download source-code so that it can be applied to data within an investigator’s own lab should be encouraged.

As algorithm-based biomarkers of aging accumulate evidence, comparative studies should be done to establish whether different measures quantify the same aging processes (Belsky et al., in press). Related questions include which measures better predict relevant outcomes in different populations at different points in the life course and whether particular risk or protective exposures modify one measure of aging but not another.

## Conclusion

Algorithm-based biomarkers of aging have the potential to advance translation of geroprotective therapies by providing clinical trials with surrogate endpoints for healthspan extension. But there is still much work to be done. Algorithm-based biomarkers of aging by construction lack specificity about the mechanism or mechanisms of aging they reflect. Instead, they seek to capture information from multiple sources to provide a summary of the organism’s rate of aging. Because of this lack of specificity regarding mechanism, algorithm-based biomarkers are only as useful as the predictions they make about healthspan. The primary empirical criterion laid out by Butler et al. (2004) more than a decade ago for candidate biomarkers of aging was that they predict age-dependent functional decline, morbidity, and mortality, and do so better than chronological age. Building from lessons learned in research on telomeres and with eye to healthspan extension as the primary target of geroprotective therapy, we suggest some additional validation criteria. Specifically, we suggest that new algorithm-based biomarkers of aging should be able to track aging-related changes in humans already by young adulthood, should show evidence of mediating risk associated with exposures known to shorten healthy lifespan, and should vary systematically in their rate of change over time such that faster change is predicted by adverse exposures and is predictive of morbidity, disability, and mortality.

## Author Contributions

DB and IS conceived the article, drafted sections of the manuscript, and critically revised the manuscript. WH conducted primary literature reviews, drafted sections of the manuscript, and critically revised the manuscript.

## Conflict of Interest Statement

The authors declare that the research was conducted in the absence of any commercial or financial relationships that could be construed as a potential conflict of interest.

## Footnotes

1 https://labs.genetics.ucla.edu/horvath/dnamage/

2 http://aging.ai/
